# Glutamatergic Enhancement Using As-Needed Dextromethorphan and Piracetam in a Stimulant-Partially Responsive Adult With ADHD: A Single-Case Report

**DOI:** 10.7759/cureus.104031

**Published:** 2026-02-21

**Authors:** Ngo Cheung

**Affiliations:** 1 Pyschiatry, Private Practice, Hong Kong, HKG

**Keywords:** adhd, adult adhd, ampa, cognition, methylphenidate (mph), nmda

## Abstract

Adults with attention-deficit/hyperactivity disorder (ADHD) frequently experience residual symptoms such as cognitive fatigue, brain fog, and reduced motivation despite optimized stimulant therapy. Emerging evidence points to disrupted dopamine-glutamate coupling and insufficient synaptic pruning as contributors to these persistent deficits, suggesting a potential role for glutamatergic modulation inspired by ketamine's rapid neuroplastic effects.

This report describes a 25-year-old man with childhood-onset ADHD who, despite trials of high-dose methylphenidate (up to 72 mg) and lisdexamfetamine (up to 80 mg), continued to report daytime lethargy, mental sluggishness, irritability, and oversleeping while on moderate-dose extended-release methylphenidate (Concerta 36-54 mg daily). After several weeks of monotherapy yielding incomplete benefit, low-dose dextromethorphan (DXM, 30 mg as needed) and piracetam (600 mg as needed) were introduced on a pro re nata (PRN, as-needed) basis, primarily on workdays with higher cognitive demands.

Within one week, the patient reported a marked and reproducible shift: elimination of afternoon tiredness, clearer thinking, reduced irritability, enhanced processing speed, and restored functional drive, with symptoms reverting on days the adjuncts were withheld. No significant adverse effects were noted.

This single-case observation suggests that low-dose dextromethorphan, through brief N-methyl-D-aspartate modulation, combined with piracetam’s α-amino-3-hydroxy-5-methyl-4-isoxazolepropionic acid (AMPA) potentiation, may enhance methylphenidate’s modest glutamate release, potentially promoting an AMPA-favorable plasticity state that could help mitigate pruning-related network inefficiencies often seen in adult ADHD. Carefully designed controlled studies are needed to better understand efficacy, optimal dosing, and long-term safety of such combinations (preliminary observation only).

## Introduction

Across the life span, attention-deficit/hyperactivity disorder (ADHD) remains common, affecting roughly three to five percent of adults who were first diagnosed in childhood and continuing to undermine executive control and motivation and affect regulation [1]. ADHD is a neurodevelopmental disorder characterized by persistent inattention and/or hyperactivity-impulsivity that interferes with functioning (DSM-5 or ICD-11 criteria). Current first-line treatment still depends on psychostimulants, principally methylphenidate and amphetamine preparations, that enhance cortical dopamine and norepinephrine signaling. Even when those medicines are carefully titrated, many patients complain of persistent "brain fog," mental fatigue, attenuated drive, and lapses of sustained attention that limit day-to-day functioning [2].

These unmet needs have shifted some of the scientific spotlight from monoamines toward glutamate. Work in animals and people now indicates that asynchronous dopamine-glutamate transmission in prefrontal networks weakens synaptic plasticity, a problem only partly corrected by stimulants [3]. Rapid-acting antidepressants such as ketamine show that pushing synapses toward an AMPA-dominant state can release brain-derived neurotrophic factor (BDNF), activate mTOR, and trigger spine formation within hours, producing behavioral benefits that outpace traditional serotonergic agents [4]. Several oral strategies aim to mimic that cascade: dextromethorphan (DXM) supplies a brief NMDA block; piracetam or other AMPA positive modulators widen downstream throughput; and supportive nutrients such as l-glutamine refill presynaptic stores [5].

Clinical hints already exist. A recent systematic review indicated that the NMDA antagonist memantine, either as a monotherapy or in conjunction with stimulants, produced modest improvements in attention and executive functioning, demonstrating favorable tolerability and supporting glutamatergic targets in both children and adults [6]. Small controlled trials of piracetam combined with methylphenidate have indicated enhanced attention and elevated response rates compared to stimulant monotherapy [7]. Notably, preclinical rodent studies show that low-dose methylphenidate (MPH) enhances NMDA-mediated synaptic currents in prefrontal cortex neurons, whereas high-dose MPH reduces glutamatergic transmission [8]. Methylphenidate itself provokes a transient glutamate surge in prefrontal cortex-a potential "push" that could be channeled through AMPA receptors if augmented by piracetam and moderated by low-dose DXM to avoid excitotoxic overshoot [8].

The present report therefore describes a 25-year-old man with chronic ADHD whose residual lethargy and cognitive sluggishness on optimal methylphenidate improved quickly and reproducibly when he used low-dose DXM and piracetam as needed. His experience offers a naturalistic illustration of how a ketamine-inspired, AMPA-centric approach may complement standard stimulant therapy in everyday practice.

## Case presentation

A 25-year-old man, followed in our Hong Kong outpatient service, carried a childhood diagnosis of ADHD, predominantly inattentive presentation with persistent residual fatigue and brain fog despite stimulant coverage (Table 1). Over the past 15 years, since his childhood diagnosis, he had tried several standard stimulants. Extended-release methylphenidate, pushed to 72 mg each morning, controlled distractibility and impulsive errors but left him anxious, mildly irritable, and underweight. Lisdexamfetamine, titrated from 30 mg to 80 mg over six weeks, produced fewer mood swings yet never quite lifted a persistent end-of-day lethargy that he described as "mental sluggishness."

**Table 1:** Treatment Timeline

Period	Medication	Dose	Observations
Childhood–2024	Various stimulants	Up to MPH 72 mg, lisdexamfetamine 80 mg	Partial control; residuals persisted
Late 2025 (restart)	Concerta (MPH)	36→54 mg daily	Stable vitals; fatigue/brain fog after four weeks
Late 2025 (+ add-ons)	+ DXM + piracetam (PRN)	DXM 15-30 mg PRN, piracetam 600 mg PRN	Rapid, reproducible gains within one week

While completing a demanding overseas work assignment in late 2025, he asked to revisit methylphenidate, hoping for crisper attention control. Concerta (methylphenidate extended-release) was restarted at 36-54 mg/day, titrated from 36 mg to 54 mg over two weeks according to clinical response; the patient had been stable on this dose for four weeks when adjuncts were added. Cardiovascular parameters remained within normal limits. With informed consent, the patient was given low-dose dextromethorphan, 15 mg tablets taken once or twice (maximum 30 mg per dose) on high-demand days, plus piracetam 600 mg capsules, usually paired with the dextromethorphan. He continued methylphenidate at the same dose. (No objective cognitive testing or laboratory assessments were performed; outcomes reflect patient self-report and clinical interview. No dissociation, agitation, or sleep disruption occurred.)

Within the first week of this PRN combination, he reported marked improvement: sustained energy through late afternoon, quicker mental processing, less irritability, and a noticeable drop in weekend hypersomnia. Benefits were reproducible, present on days he used the add-on and faded when he deliberately withheld it. No adverse effects emerged other than a brief, tolerable increase in alertness after the first few doses. The baseline stimulant regimen remained unchanged.

## Discussion

The present single-case observation adds to a growing, largely anecdotal literature hinting that glutamatergic add-ons can soften the "residual edge" often left by well-dosed stimulants in adult ADHD. In this patient, daytime fatigue, cognitive drag, and low drive persisted despite adequate trials of both high-dose extended-release methylphenidate and lisdexamfetamine. Introducing a pro re nata pairing of dextromethorphan (DXM, 15-30 mg) and piracetam (600 mg) alongside a stable Concerta regimen was followed by rapid, repeatable gains in alertness, processing speed, and mood stability, benefits not previously achieved with stimulants alone.

A plausible pharmacological explanation lies in the way these agents converge on the NMDA-AMPA balance. Therapeutic methylphenidate produces a modest, transient rise in prefrontal glutamate, mediated by dopamine D1-driven disinhibition of pyramidal cells [8]. That pulse may sharpen signal-to-noise ratios yet remain too small to trigger the full synaptic-plasticity cascade in some adults. Piracetam, acting as a positive allosteric modulator at AMPA receptors, prolongs channel open time and boosts current flow, amplifying whatever glutamate is present [9]. DXM supplies brief NMDA antagonism; at low doses, it can further lift inhibitory "brakes" on pyramidal firing while limiting calcium overload [5]. Together, the trio recreates, in miniature, the AMPA-dominant milieu thought to underlie ketamine's rapid synaptogenic effects, BDNF release, mTOR activation, and new spine formation [10]. The patient's PRN use, reserved for demanding workdays, likely helped maintain responsiveness by avoiding continuous receptor pressure.

Genetic work offers a developmental backdrop. Re-analysis of the largest ADHD GWAS shows risk variants clustering in broad synaptic-pruning pathways, complement, microglial, and cytoskeletal genes, rather than in canonical glutamatergic or monoaminergic sets [11]. The implication is that many adults with persistent ADHD carry forward networks left "over-wired" by incomplete adolescent pruning [12]. Once that window closes, adding synapses rather than trimming them may offer the more realistic route to efficiency. The pronounced response to an AMPA-centric add-on in this case fits that model: new connections, not further elimination, appear to have boosted executive capacity.

Safety is always the counterweight when manipulating glutamate. Amphetamine-class stimulants can double cortical glutamate for hours, raising theoretical excitotoxic concerns when combined with strong AMPA enhancers [13]. Methylphenidate elicits a briefer, lower-amplitude rise, and the modest DXM dose may have provided an NMDA "seat-belt" against calcium overshoot. No dissociation, agitation, or sleep disruption emerged, aligning with the generally favorable safety profile of these drugs at low doses.

Limitations are obvious. The design is n-of-1, unblinded, and reliant on self-report; placebo effects or day-to-day variability cannot be excluded. Objective cognitive testing and longer follow-up would clarify durability, tolerance, and late adverse events. Generalizability is unknown. Mechanistic interpretations remain hypothesis-generating.

Even so, the case illustrates a practical route, low-dose DXM plus piracetam for addressing stubborn cognitive-motivational symptoms in adult ADHD. The mechanistic rationale dovetails with polygenic evidence positioning the disorder as a pruning-deficient state that may benefit from synaptogenic support. Controlled trials are warranted to test whether similar regimens can reliably extend the benefits of stimulant therapy.

## Conclusions

In this naturalistic report, a 25-year-old man with adult ADHD, whose residual fatigue, brain fog, and low drive persisted despite well-titrated methylphenidate, experienced clear, repeatable improvements in daytime alertness, cognitive clarity, mood regulation, and motivation after adding low-dose dextromethorphan (≤30 mg as needed) and piracetam (600 mg), plausibly through enhanced glutamatergic (particularly AMPA-mediated) signaling and ketamine-like neuroplastic mechanisms consistent with emerging ADHD neurobiology. The observation is inherently limited by its unblinded, single-participant, self-reported, short-term design, and placebo or expectancy effects cannot be excluded; nonetheless, the rapid onset, reversibility, and tolerability observed argue that oral glutamatergic augmentation deserves formal investigation through larger controlled trials with cognitive and functional endpoints, standardized dosing, and long-term safety monitoring and that any similar off-label use in the interim should remain confined to carefully supervised clinical settings.
